# Transmission dynamics of avian influenza viruses in Egyptian poultry markets

**DOI:** 10.1038/s44298-024-00035-3

**Published:** 2024-07-02

**Authors:** Sara H. Mahmoud, Mokhtar Gomaa, Ahmed El Taweel, Yassmin Moatasim, Mina Nabil Kamel, Mohamed El Sayes, Noura M. Abo Shama, Rebecca Badra, Mona Mahmoud, Pamela P. McKenzie, Richard J. Webby, Ahmed Kandeil, Mohamed Ahmed Ali, Rabeh El-Shesheny, Ghazi Kayali

**Affiliations:** 1 Center of Scientific Excellence for Influenza Viruses, National Research Centre, Giza, 12622 Egypt; 2 Human Link, Dubai, United Arab Emirates; 3 Department of Infectious Diseases, St. Jude Children’s Research Hospital, Memphis, TN 38105 USA; 4 Present Address: Texas Biomedical Research Institute, San Antonio, TX 78227 USA

**Keywords:** Biological techniques, Genetics, Microbiology

## Abstract

Live bird markets (LBMs) are considered hotspots for Avian Influenza Viruses (AIVs). In such markets, AIVs pose threats to both poultry and public health. Within LBMs, AIVs spread through various routes, including direct contact, environmental contamination, and aerosol transmission. Unique factors in Egyptian LBMs, such as the coexistence of wild and domestic birds, increase transmission risks between birds as well as spill-overs into exposed humans. Understanding the transmission dynamics of AIVs is vital for implementing effective control measures. We conducted a study in four Egyptian LBMs located in Mediterranean coast cities from November 2021 to March 2023. In this study we tested 3,971 samples from poultry, wild birds, and the environment, out of which 692 (17.4%) were positive for AIV. Poultry exhibited a higher prevalence (42.2%) than wild birds (34.4%). Environmental samples, including water (30.8%), surfaces (17.2%), and air (18.2%), also tested positive for AIV. Diverse AIV subtypes, including H5N1, H9N2, H5/H9 co-infection, and H5N8, were detected among avian species and the environment. Temporal analysis revealed fluctuating IAV positivity rates from November 2021 to March 2023. These results emphasize the importance of continuous surveillance, resource allocation, and multisectoral collaboration to protect poultry and human health, and prevent novel influenza strains’ emergence in Egyptian LBMs.

## Introduction

Avian influenza viruses (AIVs) are commonly harbored by wild birds and can infect domestic poultry, especially waterfowl in close contact^1^. In 2020, a highly pathogenic avian influenza virus (HPAI) A(H5N1), clade 2.3.4.4b, spread among domestic and wild birds in Europe. An increased mortality in wild birds was observed in France, Belgium, the Netherlands, and Italy^2^. The virus spread to local wild birds and had undergone mutations that increased the risk of human infections. Transmission to poultry was possible as breeding birds migrate inland. HPAI H5N1 clade 2.3.4.4b transmitted from Europe into North America in late 2021 through Iceland via migratory birds^3^. In the Americas, the virus spread from Mexico to Southern Chile, mainly affecting Peruvian pelicans^2^. H5N1 Infections in mammals, likely due to contact with sick birds, were reported including a mass death event of sea lions in Peru. H5N1 infections in humans were reported from Cambodia, China, Ecuador, and Vietnam, and H5N6 infections from China and have caused severe illness and fatalities in humans^4,5^. The risk of H5 infection was assessed as low to moderate among people exposed to birds in Europe^6–8^. Highly pathogenic AIV (H5N1) was introduced into Egypt in 2005 and became endemic in poultry in 2008. Since then, many outbreaks were reported^9^. The 2.3.4.4b H5N8 virus was first detected in Egypt in wild birds in 2016 and replaced the clade 2.2.1 H5N1 viruses^10^.

Low pathogenic H9N2 avian influenza viruses typically cause mild disease in birds, often with no or minimal symptoms. However, H9N2 infection can cause a drop in egg production, and infected birds can shed and transmit the virus to other species. Moreover, H9N2 infection can cause high mortality in poultry in case of co-infection with other pathogens. In China, H9N2 viruses became endemic in commercial chicken flocks and dominant in live poultry markets since 2016^11^. Over 100 human cases of H9N2 virus were reported, with more than 50 cases occurring after the COVID-19 outbreak^2,12^. A seroprevalence rate of 11.2% against H9N2 AIVs among healthy occupational workers was detected in several provinces of China during 2014–2016^13,14^. In Egypt, H9N2 AIV are endemic in poultry and are continuously circulating in LBM despite the use of avian influenza vaccines. Reassortant H9N2 viruses were detected^15^. Co-circulation of H5 and H9N2 viruses enhances the likelihood of genetic reassortment, which might increase the zoonotic potential.

Handling and transporting infected birds in markets contribute significantly to avian influenza transmission. Close contact between humans and infected birds poses risks to market workers and visitors^16^. Activities like capturing, restraining, and moving infected birds bring humans in direct contact with them. Handling infected birds without proper personal protection and hygiene practices can directly transmit avian influenza viruses to humans. Human-to-human transmission of avian influenza, specifically H5N1 and H7N9 strains, is possible but limited. Live bird market (LBM) settings, where birds and humans closely interact, provide an environment for the virus to potentially adapt and undergo genetic mutations that might improve virus infectivity and transmissibility in humans. This raises concerns about a potential pandemic if the virus becomes easily transmissible between people^17^.

When different bird species come in close proximity, especially in live bird markets selling both poultry and trapped wild birds, there is a higher risk for cross-species transmission of viruses. Co-existence of infected and susceptible birds in markets provides opportunities for the virus to spread and infect new hosts^18–20^. In Egyptian markets, workers face occupational hazards related to avian influenza viruses, including direct contact with infected birds or their bodily fluids, exposure to contaminated surfaces and equipment, and inhalation of airborne particles carrying the virus^21,22^.

We have been conducting routine active surveillance of avian influenza viruses in LBM and migratory wild birds in Egypt since 2009 to monitor influenza viruses with pandemic potential at the human–animal interface and identify the genetic and antigenic characteristics of influenza A viruses that were introduced into Egypt. We reported the first detection of H5N1 clade 2.3.4.4b viruses in wild birds and domestic ducks from live bird market samples from 2021-2022 in Egypt and showed that the virus could be able to infect and cause disease in mammals^9^, which required continuous monitoring of AIV in Egypt.

Here, we provide an update on the epizootiology and subtypes of AIVs circulating in Egyptian live bird market. We conducted a study in four Egyptian LBMs located in Mediterranean coast cities from November 2021 to March 2023. In this study we tested 3,971 samples from poultry, wild birds, and the environment to understand the transmission dynamics of AIVs and potential environmental contamination.

## Materials and Methods

### Avian influenza surveillance

Active surveillance for AIVs was conducted in four Egyptian live bird markets located in Mediterranean coast cities (Baltim, Damietta, Port-said, and Rasheed) from November 2021 to March 2023. A unique feature of those markets is that trapped wild birds are sold live alongside live domestic poultry. Biosecurity measures in these markets were relatively low, increasing the risk of contact between commercial poultry and wild birds. Each of the LBMs was visited once per month to capture seasonal variations in AIV prevalence, ensure sampling adequacy, enhance detection sensitivity, and optimize resource utilization. Oropharyngeal and cloacal swabs were collected from domestic poultry and wild birds in a viral transport medium.

### Environmental sample collection

Sample collection was carried out in the slaughter zone and sale zone of LBMs to encompass areas with potential high viral loads and diverse avian interactions. Given the unique dynamics of Egyptian LBMs, where a variety of avian species, including both wild and domestic birds, coexist in close proximity, the choice of surfaces for sampling was crucial. Surfaces such as cages, feeders, and slaughtering tables were selected based on their direct or indirect contact with live poultry. These surfaces represent critical points of interaction between birds and environmental elements within the market, thereby serving as potential fomites for virus transmission. The variation in the number of samples per visit was determined based on the diversity of surface types and their respective exposure to poultry. For instance, surfaces directly in contact with live poultry, such as feeders and cages, were sampled more extensively due to their higher likelihood of harboring viral contaminants. In contrast, surfaces with indirect poultry contact, such as slaughtering tables, were sampled to a lesser extent but still included to assess potential environmental contamination during market operations. This approach allowed us to capture a comprehensive snapshot of environmental viral dissemination within LBMs while optimizing resource allocation and sampling efficiency. In this study, where AIVs are known to spread through various routes, including aerosol transmission, determining the detection limit of the air sampler is crucial for assessing the risk of viral dissemination in the market environment. The ability to detect viral particles at low concentrations allows us to monitor the presence of AIVs in the air, which can serve as an early warning system for potential outbreaks or increased transmission rates within the LBMs.

Air sampling was performed with a Coriolis Micro biological air sampler (BERTIN TECHNOLOGIES, France). The cyclonic technology, paired with a high airflow rate of up to 300 L/min, allows reliable virus collection. The detection limit of 100 genomes/m^3^ refers to the minimum concentration of viral genomes in the air that can be reliably detected using the Coriolis Micro biological air sampler employed in our study. This limit was determined based on the manufacturer’s specifications and performance characteristics of the sampler. The Coriolis Micro sampler utilizes cyclonic technology coupled with a high airflow rate of up to 300 L/min, which enables efficient collection of airborne particles, including viruses.

According to the manufacturer’s instructions, a concentration of only 100 genomes/m^3^ is detectable in samples collected in an infection medium (medium containing 4% Bovine Serum Albumin (BSA)). A collection cone, pre-filled with an infection medium, is placed on the biological air sampler. Once initiated, Coriolis Micro generates a vortex in the collection cone. Viruses or microorganisms are centrifuged onto the inner walls of the cone and are separated from the air. The sample is then ready for collection in cryovials for RNA extraction. Disinfection was performed by rinsing the collection vessel with water followed by contact with 70% absolute alcohol for one minute and one other water rinse.

Sterile swabs were used to wipe different spots in live bird markets. Surface types included cages, feeder (feed holder for poultry to eat from most often a bowl or trough), and tables used during the slaughtering of some poultry to be sold inside the market. Most of surfaces were in direct contact with the poultry, where live poultry could potentially touch the surface (e.g., Feeder or cages), or in indirect contact with the live poultry, (e.g., slaughtering spots). Different surface types were sampled at each market for a total of five samples per visit with the maximum possible amount of exposed surface area (i.e., area exposed to poultry) wiped on each surface (varied for each surface). Individual wipes were placed in vials containing 5 ml of viral transport media and frozen (–80 °C) until testing.

Water sampling from waterers in LBMs is crucial due to its potential role in AIV transmission dynamics. Waterers serve as essential sources of drinking water for both domestic poultry and wild birds within LBMs. Given the communal nature of water sources in these settings, they can become reservoirs for AIVs shed by infected birds. Therefore, by specifically targeting waterers, we aimed to capture any potential viral contamination present in these critical environmental reservoirs.

Water samples were collected mostly from Waterer (water holder for poultry to drink; most often a nipple waterer or bowl), 5 ml were collected directly from each waterer and five samples were collected per each side and frozen until viral RNA extraction to adequately assess the spatial distribution of AIVs within LBMs. LBMs are dynamic environments where birds move freely and interact with various surfaces. By collecting multiple samples from different locations within each side of the market, we aimed to capture the variability in viral contamination across different areas. This approach provides a more comprehensive understanding of AIV prevalence and distribution within LBMs, allowing for targeted interventions to mitigate transmission risks.

Nasal swabs and hand wipes were collected from market workers to detect any infection in exposed workers after completing written informed consent. Handwipes were collected using 2×2 inch gauze strips saturated with sterile viral transport media. All collected samples were kept on ice until received in the laboratory then stored at -80 °C until use. A total of 1880 bird samples, 1660 human samples, and 431 environmental samples were analyzed. Field data, including sample date and type, species, health status, age, and sex, were recorded. This study was approved by the Ethics Committee of the National Research Centre, Egypt and the Institutional Review Board (protocol number 14 155), and the Institutional Animal Care and Use Committee of St. Jude Children’s Research Hospital, USA.

### Sample screening and virus isolation

Viral RNA was extracted from the original swabs, collected air, water, surface samples, and hand wipes using the MagNA Pure 96 platform and the KingFisher Flex instrument (Thermo Fisher Scientific, Rocklin, CA, USA), according to the manufacturer’s instructions. The extracted RNA was then tested for AIVs using real-time RT-PCR (rRT-PCR) for the presence of AIV targeting the universal M-gene^23^. Samples with a Ct value of 35 or lower were considered positive. Positive samples in the influenza matrix gene rRT-PCR were individually inoculated into the allantoic cavity of specific pathogen-free (SPF) embryonated chicken eggs. The eggs were incubated at 37 °C for 48-72 hours. Allantoic fluid was collected after chilling the eggs at 4 °C overnight and used for a hemagglutination assay (HA) with 0.5% chicken erythrocytes, following the protocols of the World Health Organization (WHO). Samples with positive HA titers were then subtyped and sequenced as previously described^24,25^.

### Sample sequencing

Viral RNA from allantoic fluid was used to synthesize the first cDNA strand through reverse transcription using the SuperScript IV first-strand synthesis kit and the Uni12 influenza primer. Multiplex PCR of all eight gene segments was performed using Phusion high-fidelity DNA polymerase and Uni12/13 primers. The PCR products were purified, and sequencing library preparation was conducted with Illumina’s Nextera XT DNA Sample Preparation Kit. The amplicons were then sequenced on Illumina’s MiSeq platform using the paired-end approach. The eight full segments of each H5N8 virus were assembled using CLC Genomics Workbench, version 21. To control the risk of PCR contamination, all laboratory procedures were conducted in a dedicated PCR facility with separate workstations, physical barriers were considered, positive and negative controls were used, physical separation of pre- and post-PCR areas, and decontamination protocols were followed.

### Statistical analysis

The Chi-square test was used to perform statistical comparisons. A *p* value of < 0.05 was considered to indicate statistical significance. Analysis was performed using Microsoft Excel built-in functions and tools for basic statistical analyses.

## Results

### Prevalence of AIVs in LBMs and wild birds

The distribution of the collected samples and influenza A detection rate within each category are presented in Table 1. The table shows the number and percentage of collected samples that tested positive for Influenza A virus and provides the corresponding p-values to indicate the statistical significance of observed differences. **A**cross various sample types, including avian, environmental, and human, the prevalence of Influenza A virus varied significantly (*p* < 0.001). The collected samples included 608 oropharyngeal (32.3%) and 608 cloacal samples (32.3%) from domestic poultry intended for human consumption, 332 oropharyngeal (17.7%) and 332 cloacal samples (17.7%) from wild bird species sold in the live bird markets, 197 surface samples (45.7%), 201 water samples from drinking water troughs (46.6%), 33 air samples (7.7%), and 830 human nasal swabs and 830 wipes from individuals working in the market.

**Table 1:** Comparison of Influenza A positivity rate by variable and sample type

Variable	Sample type	Collected samples no. (%)	IAV positive samples no. (%)	*P* values
**Avian samples**	Poultry (Oropharyngeal)	608/1880 (32.3%)	160/608 (26.3%)	<0.001 (highly significant)
	Poultry (Cloacal)	608/1880 (32.3%)	97/608 (15.9%)	
	Wild bird (Oropharyngeal)	332/1880 (17.7%)	65/332 (19.6%)	
	Wild bird (Cloacal)	332/1880 (17.7%)	49/332 (14.8%)	
**Environment**	Surface	197/431 (45.7%)	34/197 (17.2%)	<0.001 (highly significant)
	Water	201/431 (46.6%)	62/201 (30.8%)	
	Air	33/431 (7.7%)	6/33 (18.2%)	
**Human**	Human swabs (Males/Females)	830/1660 (50%)	113/830 (13.6%)	0.408 (not significant)
	Human wipes (Males/Females)	830/1660 (50%)	106/830 (12.8%)	
**Total Avian**	1880			
**Total Poultry**	1216			
**Total Wild**	664			
**Total Env**.	431			
**Total Human**	1660			

Out of the 3971 samples analyzed, influenza A virus (IAV) was detected in 692 (17.4%) samples. Out of the 608 oropharyngeal and 608 cloacal poultry samples, 160 (26.3%) and 97 (15.9%) samples tested positive for IAV respectively (*p* value < 0.001). Additionally, out of 332 oropharyngeal and 332 cloacal wild bird samples collected, 65 (19.6%) and 49 (14.8%) samples tested positive for IAV respectively (p-value < 0.001). It is worth noting that while nearly all samples (99%) were collected from apparently healthy birds, IAV was detected in 17.4% of them. The detection rate of influenza was found to be higher in poultry intended for consumption compared to wild birds (42.2% vs 34.4%, *p* < 0.001).

Regarding environmental samples, 62 (30.8%) out of 201 water samples, 34 (17.2%) out of 197 surface samples, and 6 (18.2%) out of 33 air samples tested positive for IAV (*p* value < 0.001). From human samples, a total of 113 (13.6%) nasal swabs and 106 (12.8%) hand wipes tested positive for IAV. The *p* value for this comparison is 0.408, indicating that the difference is not statistically significant.

Table 2 provides the total number of AIV-positive samples, subtypes, and isolates presented by different species and environmental samples.

**Table 2:** Number of IAV positive samples, subtypes, and isolates presented by species

Host common name	Species	Total samples	IAV positive samples	Subtypes				Total subtypes	Isolates
				H5N1	H5N8	H9N2	H5/H9		
Black-legged kittiwake	Rissa tridactyla	6	2					0	
Cattle egret	Bubulcus ibis	2	0					0	
Chicken	Gallus gallus domesticus	688	152	1		48	1	50	23 (H9N2)
Common myna	Acridotheres tristis	2	0					0	
Common pochard	Aythya ferina	62	7		1			1	
Common quail	Coturnix coturnix	140	27			4		4	1 (H9N2)
Common coot	Fulica atra	30	1					0	
Duck	Anas sp.	104	33	4	2		1	7	
European turtle dove	Streptopelia turtur rufescens	10	0						
Eurasian golden oriole	Oriolus oriolus	14	1						
Garganey	Anas querquedula	178	36	3		1		4	1 (H9N2)
Hoopoe	Upupa epops	2	0						
Mallard duck	Anas platyrhynchos	44	6						
Moorhen	Gallinula sp.	134	14						
Muscovy duck	Cairina Moschata	10	8	6				6	4 (H5N1)
Northern shoveler	Anas clypeata	156	33						
Pigeon	Columba sp.	164	15	1				1	
Northern pintail	Anas acuta	86	21	1				1	1(H5N1)
Purple swamphen	Porphyrio porphyrio	10	7						
Ruff	Philomachus pugnax	16	1						
Saker falcon	Falco cherrug	10	2			1		1	1 (H9N2)
Turkey	Meleagris sp.	10	5			2		2	2 (H9N2)
Eurasian wigeon	Anas penelope	2	0						
**Environment**									
Surface		197	34	1		4		5	1 (H5N1), 4 (H9N2)
Water		201	62			7		7	4 (H9N2)
Air		33	6						
**Total number of samples**		**2311**							
**Negative**		**1838**							
**Positive**		**473**							

Black-legged kittiwake had two positive samples out of six collected samples, but no specific subtypes or isolates were reported. Cattle egret, common myna, European turtle dove, hoopoe, and Eurasian wigeon did not show positive AIV samples. Chicken samples had a high number of positive cases, with 152 out of 688 samples testing positive for Influenza A. One sample was identified as subtype H5N1, 48 samples were identified as H9N2, one sample identified as H5/H9 coinfection, and 23 H9N2 isolates were obtained. Common pochard had seven positive samples out of 62, with one H5N8 subtype. Common quail had 27 positive samples out of 140, including four samples of subtype H9N2, and one isolate of subtype H9N2. Common coot had only one positive sample out of 30. Duck samples showed 33 positive cases out of 104, with four samples identified as subtype H5N1, two samples of subtype H5N8, and one sample of subtype H5/H9. Among the analyzed avian samples, the Eurasian golden oriole showed one positive sample out of 14 tested. The Garganey had 36 positive samples out of 178, with three samples identified as subtype H5N1, one sample identified as subtype H9N2, and one isolate of H9N2 subtype. The Mallard duck had six positive samples out of 44, while the Moorhen had 14 positive samples out of 134 collected. The Muscovy duck showed 8 positive samples out of 10, with six samples identified as subtype H5N1. Four isolates of subtype H5N1 were obtained from the Muscovy Duck samples. The Northern shoveler had 33 positive samples out of 156, while the Pigeon had 15 positive samples out of 164, with one sample identified as subtype H5N1. The Northern pintail showed 21 positive samples out of 86, with one sample identified as subtype H5N1. One isolate of subtype H5N1 was obtained from the Pintail samples. The Purple swamphen exhibited 7 positive samples out of 10, while the Ruff had only one positive sample out of 16. The Saker falcon had two positive samples out of 10, with one sample identified as subtype H9N2. One isolate of subtype H9N2 was obtained from the Saker falcon samples. The Turkey had five positive samples out of 10, with two samples identified as subtype H9N2. Two isolates of subtype H9N2 were obtained from the Turkey samples. These findings demonstrate the presence of Influenza A in various avian species, highlighting the diversity of subtypes and isolates circulating in the studied samples.

Among the environmental samples, 34 out of 197 surface samples tested positive for Influenza A, with one sample identified as subtype H5N1 and four identified as H9N2; five isolates were obtained (one of subtype H5N1 and four of subtype H9N2). Water samples had 62 positive cases out of 201 with seven samples of H9N2 subtype; four isolates of subtype H9N2 were obtained. Air samples showed six positive cases out of 33.

The data obtained from these avian and environmental samples provide valuable insights into the diversity of circulating AIV subtypes and isolates and highlight the environmental contamination by various IAV subtypes across the LBMs.

### Seasonality of avian influenza viruses in LBMs and environments

**Fig. 1:**
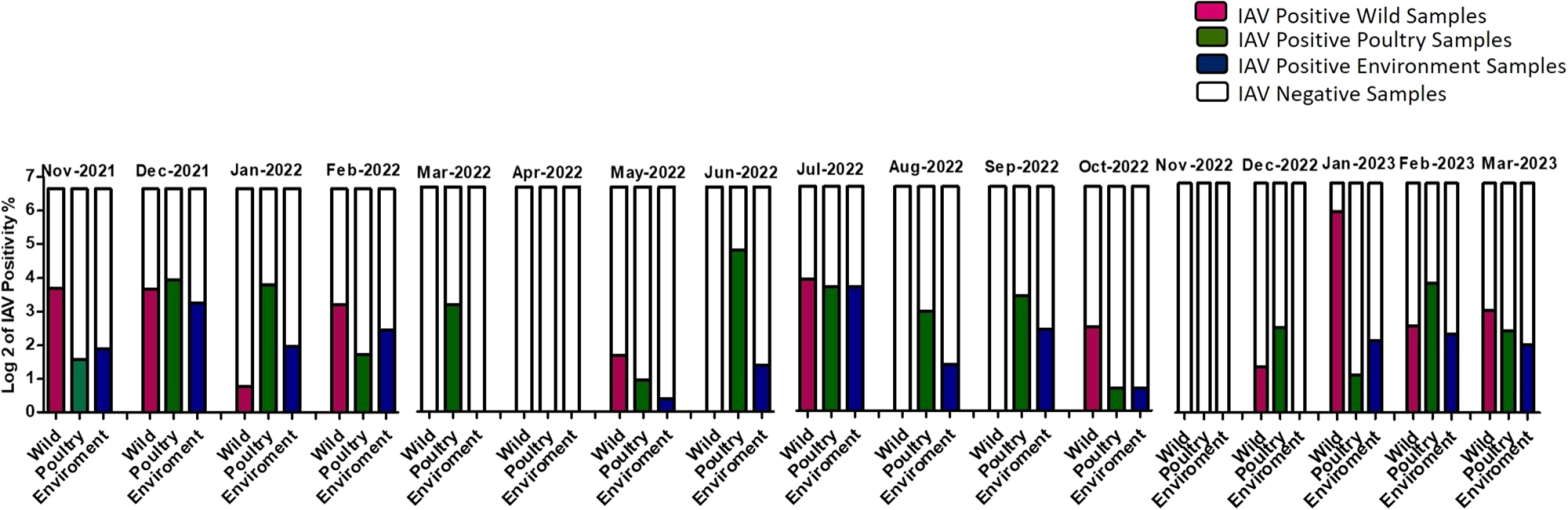
Positivity rate of IAV in poultry, wild birds, and environment by month during active AIV surveillance from November 2021 to March 2023 in Egyptian LBM. IAV-positive wild bird samples are represented in pink, IAV-positive poultry samples are represented in green, IAV-positive environment samples are represented in blue, and IAV-negative samples are represented in white.

The monthly positivity rate of IAV in wild birds, poultry, and the environment from November 2021 to March 2023 is presented in Fig. 1. Among wild bird samples, the positivity rate of IAV fluctuated from 0% to 56.9% during the study period. The highest positivity rate was observed in January 2023, while the lowest was recorded in January 2022. No positive samples were detected in April, June, August, September, and November 2022. The positivity rate of IAV in poultry ranged from 0% to 27.4%. The highest positivity rate was observed in June 2022, while the lowest was recorded in October 2022. Positive samples were not detected in April and November 2022. The positivity rate of IAV across environmental samples ranged from 0% to 12.8%. While the highest positivity rate was observed in July 2022, the lowest was recorded in May 2022. No positive samples were detected in April, November, and December 2022. These percentages provide insights about the circulation of influenza A virus in the respective sample types (wild birds, poultry, and the environment) throughout the study period.

Additionally, data analysis revealed potential transmission routes among poultry, wild birds, and the environment. Periods with relatively high positivity rate of IAV in poultry indicate possible transmission within poultry populations. Similarly, wild birds AIV positivity rate exhibited variability throughout the study period, suggesting potential transmission within wild bird populations.

The positivity rate of IAV in water, surface, and air samples also fluctuated, indicating the presence of the virus in the environment and potential environmental contamination. Overall, the findings indicate a fluctuating presence of IAV among poultry, wild birds, and the environment during the specified time period. These results suggest the possibility of viral transmission and circulation among these different sources.

### Variations of the AIV positivity rates and subtypes among different sample types

**Fig. 2:**
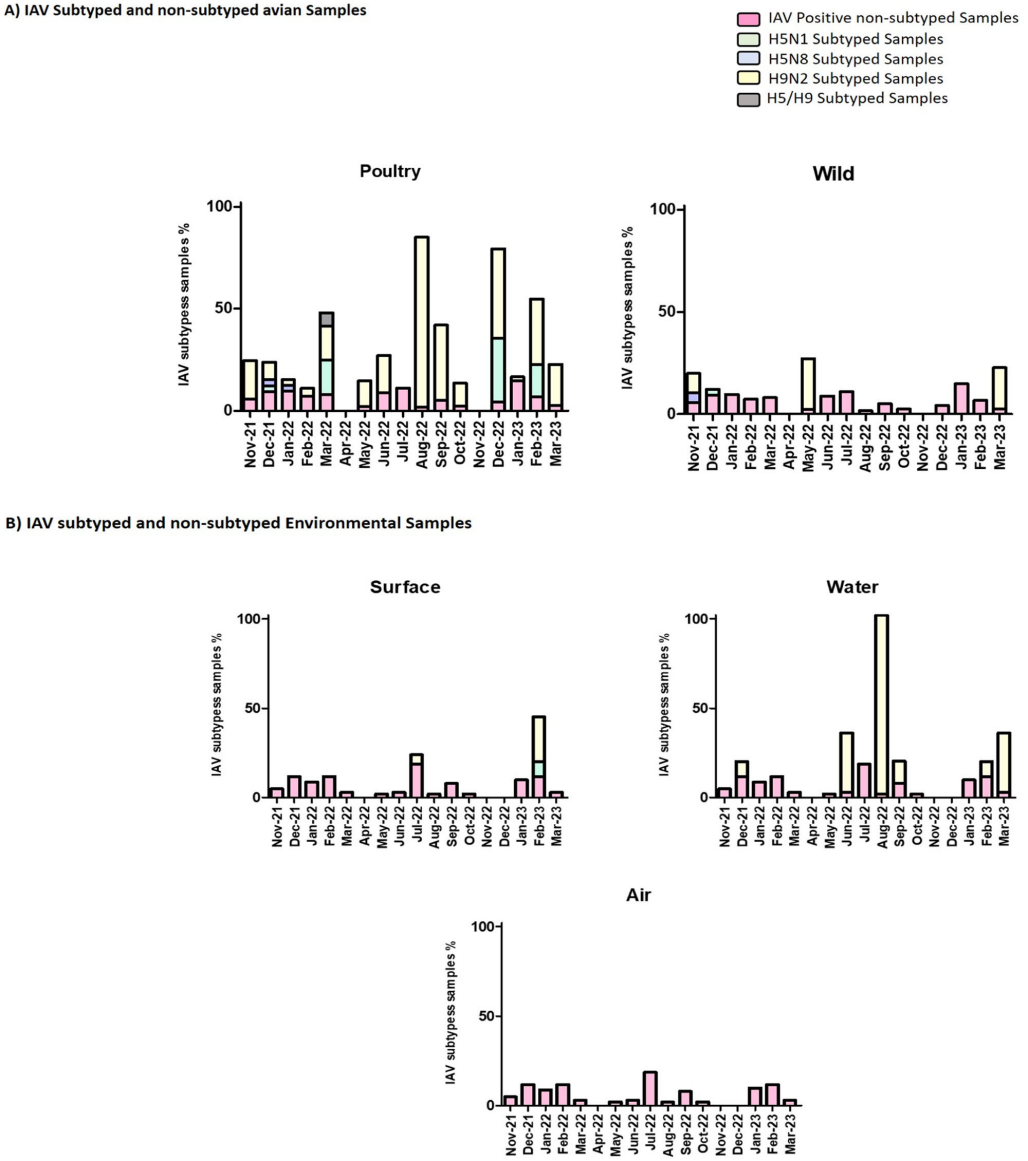
Percentage of AIV positive non-subtyped and subtyped (H5N1, H5N8, and H9N2) in avian and environmental samples by month during surveillance from November 2021 to March 2023. **A** IAV subtyped and non-subtyped in avian samples, in poultry and wild birds; **B** IAV subtyped and non-subtyped in environmental samples: surface, water, and air. IAV positive non-subtyped samples are represented in light red, H5N1 subtyped samples are represented in light green, H5N8 subtyped samples are represented in light blue, H9N2 subtyped samples are represented in light yellow, and H5/H9 subtyped samples are represented in grey.

The presence and prevalence of different IAV subtypes are illustrated in Fig.2. The percentage of total avian-positive influenza samples varied across the months, ranging from 1.62% to 14.82%, indicating the presence of viral infection in poultry and wild birds within the market. The percentage of total environmental positive IAV samples varied across the months, ranging from 1.96% to 18.63%, indicating the presence of viral contamination in the market environment.

#### Poultry samples

In poultry samples, the H5N1 subtype was detected in December 2021, March 2022, December 2022, and February 2023 with prevalence rates of 2.86%, 16.67%, 31.25%, and 16.0%, respectively, among the positive samples. H5N1 is a highly pathogenic strain of avian influenza associated with severe illness and high mortality rates in birds and, in rare cases, in humans. The presence of H5N1 in the poultry samples indicates its circulation within the market environment during those specific months. The H5N8 subtype was detected in December 2021 and January 2022 with prevalence rates of 2.857% and 2.778%, respectively. The H9N2 subtype was detected in the poultry samples in November 2021, December 2021, January 2022, February 2022, March 2022, May 2022, June 2022, August 2022, September 2022, October 2022, December 2022, January 2023, February 2023, and March 2023, with prevalence rates ranging from 2.78% to 83.33% among the positive samples. H9N2 is a low pathogenic strain of avian influenza that can infect various bird species, including poultry. The presence of H9N2 in these poultry samples indicates its continuous circulation and potential transmission within the market environment throughout the surveillance period. The H5/H9 subtype was detected in March 2022 with a prevalence rate of 6.667%.

#### Wild samples

In wild bird samples, H5N1 subtype was only detected in December 2021 with 2.9% prevalence rate. The H5N8 subtype was detected in wild avian samples in November 2021, with a prevalence rate of 4.76% among the positive samples. H5N8 is a highly pathogenic strain of avian influenza that has been associated with severe illness and high mortality rates in birds. The presence of H5N8 in the wild avian samples indicates its circulation within the market environment during that specific month. The H9N2 subtype was detected in the wild avian samples in November 2021, May 2022, and March 2023, with prevalence rates ranging from 9.52% to 25% among the positive samples. H9N2 is a low pathogenic strain of avian influenza that can infect various bird species, including wild birds. The presence of H9N2 in these wild avian samples indicates its sporadic presence and potential transmission within the market environment during those specific months. The H5/H9 subtype was not detected in any of the wild avian samples.

These results highlight the importance of ongoing surveillance and monitoring of IAV subtypes in wild avian populations within Egyptian markets to understand the prevalence, circulation, and potential risks associated with different strains. It also emphasizes the need for appropriate biosecurity measures and interventions to prevent the transmission and spread of avian influenza among wild bird populations and to minimize the risk of zoonotic transmission to both animals and humans.

#### Surface samples

In surface samples, no H5N1 or H5N8 subtypes were detected during the entire period except only for H5N1 was detected in February 2023 with a prevalence of 8.3%. However, there was a detection of the H9N2 subtype in July 2022 and February 2023, with a prevalence of 5.26% and 25% respectively among the positive samples. No H5/H9 subtype was identified in any of the surface samples during the specified timeframe.

Overall, the graph indicates that while positive IAV samples were found on surfaces in Egyptian markets during surveillance, only the H9N2 subtype was detected, and it was present in a relatively low proportion in July 2022 and high proportion in February 2023 in addition to H5N1 that was detected with 8.3% prevalence in February 2023. It is important to note that the absence of specific subtypes (H5N8 or H5/H9) in the surface samples does not necessarily imply their absence in other sample types or at different time points.

#### Water samples

No water samples tested positive for H5N1 or H5N8, which are highly pathogenic strains associated with avian influenza. However, the H9N2 subtype was detected in December 2021, June 2022, August 2022, September 2022, February 2023, and March 2023, with prevalence rates of 8.33%, 33.33%, 100%, 12.5%, 8.3%, and 33.33%, respectively, among the positive samples. The H5/H9 subtype was not detected in any of the water samples throughout the surveillance period. It’s important to note that the absence of certain subtypes in the water samples does not imply their absence in other sample types or locations within Egyptian markets. The results highlight the need for continued surveillance and monitoring to understand the prevalence and distribution of different IAV subtypes in the water within the market environment.

#### Air samples

No specific IAV subtypes (H5N1, H5N8, H9N2, or H5/H9) were detected in any of the air samples throughout the entire period. It is important to note that the data presented in the graph only pertains to the specified time period and does not indicate the absence of these subtypes in other sample types or during different timeframes. Several factors may contribute to the absence of specific IAV subtypes in air samples.

### Limitations and implications

While our study provides valuable insights into the prevalence and diversity of AIVs in Egyptian LBMs, it is crucial to acknowledge certain limitations that may impact the interpretation of our findings and their broader implications.

One significant limitation of our study is the bias inherent in sampling apparently healthy birds. Detecting viruses primarily in healthy birds may not fully represent the true prevalence of AIVs in LBMs or accurately assess the potential risk to public health. By focusing on apparently healthy birds, we may underestimate the overall prevalence of AIVs, as sick birds are more likely to exhibit viral shedding and be targeted for sampling in other studies. Additionally, the presence of AIVs in asymptomatic carriers among apparently healthy birds emphasizes the silent threat posed by these carriers in LBMs. The implications of this sampling bias are multifaceted. First, relying solely on samples from apparently healthy birds may lead to an incomplete understanding of AIV transmission dynamics within LBMs. The true extent of viral circulation and transmission pathways, particularly from asymptomatic carriers to susceptible hosts, may be underestimated. Consequently, the efficacy of control measures and intervention strategies based on this incomplete understanding may be compromised.

Moreover, the method used for air sampling, while effective for detecting airborne particles carrying viruses, may have limitations in capturing specific IAV subtypes. The shedding dynamics of different IAV subtypes by infected birds and environmental sources may vary. Certain subtypes may be shed in higher quantities through respiratory secretions or fecal matter, making them more likely to be detected in environmental samples such as water or surfaces compared to air samples. Airborne viruses may undergo degradation or loss of infectivity over time due to environmental factors such as temperature, humidity, and exposure to ultraviolet (UV) radiation from sunlight. The stability of specific IAV subtypes in the air may differ, affecting their detectability in air samples.

Furthermore, the underestimation of AIV prevalence in LBMs could have implications for public health risk assessment. Failure to accurately assess the prevalence of AIVs, especially in environments where humans come into close contact with infected birds, may underestimate the potential for zoonotic transmission and the risk of novel influenza strain emergence. This underscores the importance of considering not only the prevalence of AIVs but also their distribution and transmission dynamics in LBMs to adequately assess public health risks and inform preventive measures.

In addition, the sampling bias may limit the generalizability of our findings to other LBMs or regions with different environmental, ecological, or operational characteristics. Variations in market settings, biosecurity measures, poultry management practices, and wild bird populations may influence the prevalence and transmission dynamics of AIVs. Therefore, caution should be exercised when extrapolating our findings to broader contexts without considering these factors.

While our study provides valuable insights into AIV prevalence and diversity in Egyptian LBMs, the limitation associated with sampling bias underscores the need for future research to adopt more comprehensive sampling strategies that account for both apparently healthy and clinically ill birds. Addressing this limitation will improve our understanding of AIV transmission dynamics, enhance risk assessment capabilities, and inform more effective control and prevention strategies to mitigate the threat of avian influenza in LBMs and safeguard both animal and public health.

## Discussion

AIVs are continuously circulating in Egyptian live bird markets, posing risks to the poultry industry and public health. Live bird markets in Egypt facilitate the trade and allow the interaction of various bird species, which increases potential AIVs transmission^26^. Monitoring and surveillance of avian influenza viruses in Egyptian live bird markets are crucial to track the presence of AIVs and other avian pathogens. Active surveillance programs allow regular collection of samples from different sources in the markets, including poultry, wild birds, and the environment, such as water, surfaces, and air^27^.

AIVs in Egyptian markets spread through direct contact, environmental contamination, and aerosol transmission. Infected birds transmit the virus through pecking, feeding, or mating. AIVs are shed through respiratory secretions, feces, saliva, and ocular discharge and spread through coughing, sneezing, and normal respiration. Respiratory droplets and dust particles carry the virus infecting birds that inhale them. Contaminated surfaces and equipment within markets play a role in transmission when infected birds come into contact with them. Therefore, this environmental contamination exposes susceptible birds to infection. These routes apply to avian markets worldwide but specific factors in Egyptian markets, such as environmental conditions and the coexistence of domestic and wild birds, increase transmission risks^28,29^.

The study was conducted in four Egyptian LBMs located in Mediterranean coast cities. This choice of study sites was intentional to capture the real-world scenario prevalent in many LBMs in Egypt. Monthly active surveillance for AIVs was conducted over a span of 16 months, from November 2021 to March 2023, to assess the prevalence and circulation of AIVs among poultry, wild birds, and the environment within these LBMs. This prolonged surveillance period allowed us to capture seasonal variations and assess the impact of low biosecurity measures over time.

Variation in AIV positivity rates among sample types (higher prevalence of AIV in poultry compared to wild birds) can be attributed to several factors. Poultry in LBMs are often intensively raised in proximity, facilitating rapid virus transmission. Conversely, wild bird populations may have lower densities and less frequent interactions, reducing the likelihood of virus spread. Furthermore, differences in immune status and susceptibility between poultry and wild birds may contribute to varying positivity rates. Poultry in LBMs are often exposed to stressors that compromise immune function, making them more susceptible to AIV infection compared to wild birds in their natural habitats. The observed variations in AIV positivity rates among different sample types underscore the multifactorial nature of AIV transmission within LBMs. By considering temporal dynamics, species-specific transmission patterns, and market hygiene practices, we can better understand and mitigate the risks associated with AIV circulation in Egyptian LBMs. Further research and surveillance efforts are warranted to elucidate the complex interactions driving AIV transmission and inform targeted control strategies.

Environmental samples, including water, surfaces, and air, also tested positive for AIV, albeit at lower rates than poultry suggesting environmental contamination in LBMs that can serve as a reservoir for viral persistence and transmission. Factors such as inadequate sanitation practices and overcrowding may exacerbate environmental contamination, leading to sporadic AIV detection. The fluctuating positivity rates in environmental samples may reflect seasonal variations in LBM activities, such as increased trade during certain periods or changes in environmental conditions conducive to viral survival. The presence of H9N2 subtype in both surface and water samples suggests that infected birds shed the virus into the environment, contaminating surfaces and water sources commonly used by both poultry and wild birds. The coexistence of wild and domestic birds in LBMs enhances transmission risks, as close contact between species facilitates viral spread. The detection of H9N2 in surface and water samples underscores the risk of viral transmission to both avian species and potentially exposed humans. Infected surfaces and water sources serve as reservoirs for the virus, posing continuous threats to the poultry industry and public health.

The fluctuating positivity rates observed over the surveillance period suggest dynamic AIV transmission dynamics within LBMs. Seasonal factors, such as changes in bird migration patterns or environmental conditions, may influence virus circulation. Additionally, temporal variations in market activities, such as restocking of poultry or influx of migratory birds, could impact AIV prevalence. Cold, dry conditions support AIV survival and transmission, while warmer temperatures reduce viability. Higher AIV rates in colder months like January and February in the Mediterranean region align with this trend^30^. Understanding these temporal patterns is crucial for targeted surveillance and intervention strategies.

The diversity of AIV subtypes and isolates detected across avian species highlights the complex transmission dynamics within LBMs. Certain bird species may act as reservoirs for specific AIV subtypes, facilitating interspecies transmission. The presence of highly pathogenic AIV subtypes, such as H5N1 and H5N8, underscores the potential for zoonotic spillover events and the need for vigilant surveillance and control measures.

**Fig. 3:**
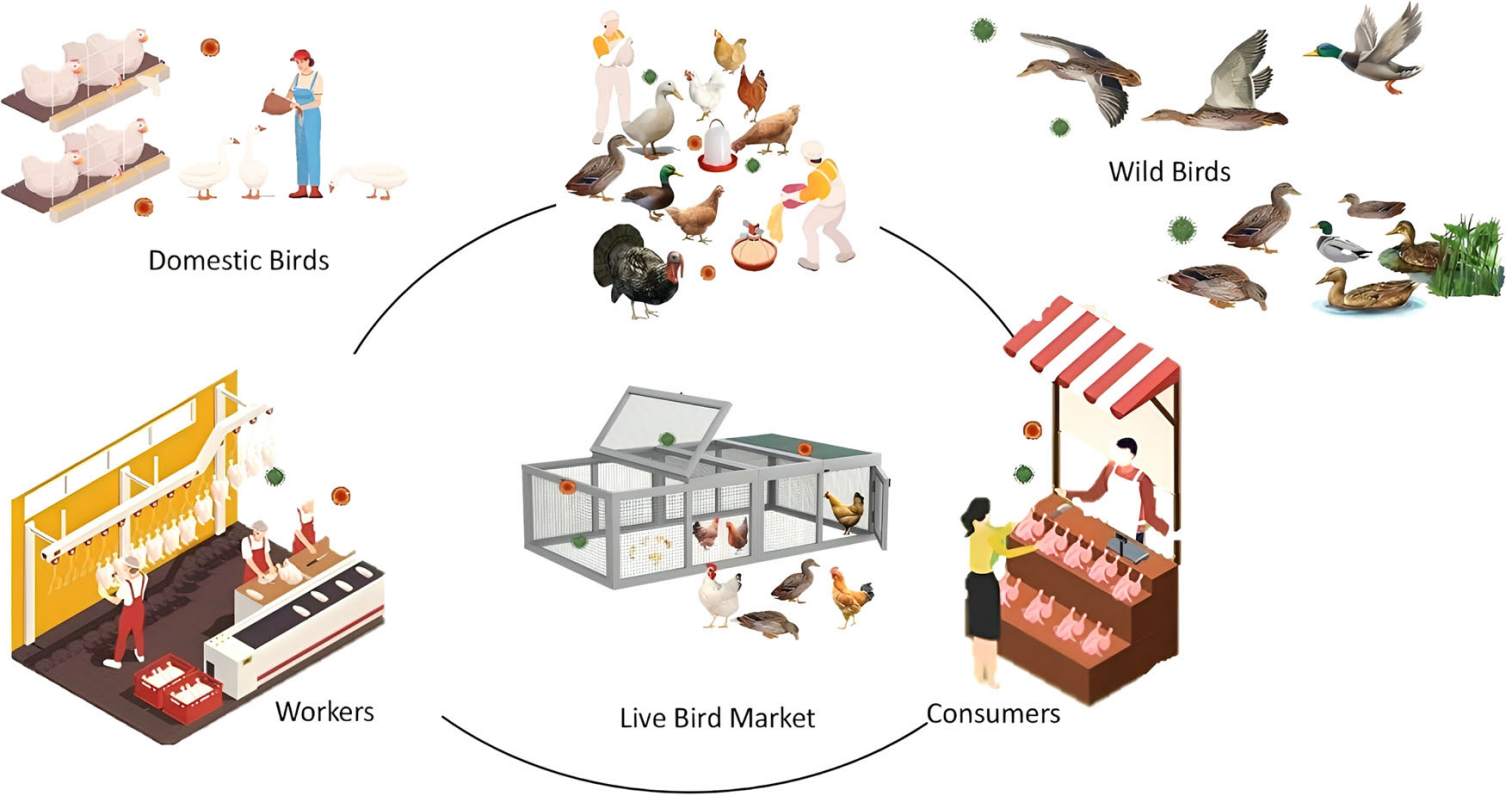
Transmission pathways of avian influenza viruses among poultry and wild birds in shared environments, and human interactions in LBM. Avian influenza viruses in wild birds are represented in green circles and avian influenza viruses in domestic birds are represented in red circles.

Variations in AIV positivity rates among different sample types may also reflect differences in market hygiene and biosecurity practices. Inadequate biosecurity measures, such as poor sanitation or insufficient quarantine procedures, can increase virus transmission within LBMs. Direct contact between humans and infected birds, as well as environmental contamination, were facilitated by the lack of stringent biosecurity protocols. The transmission routes of AIVs in environments where both poultry and wild birds coexist are summarized in Fig.3. The main pathways include the transmission of AIVs through shared drinking water sources, airborne dissemination, and contamination of various surfaces. The higher prevalence of AIVs detected in poultry intended for consumption compared to wild birds underscores the role of biosecurity in mitigating transmission among poultry populations. The close confinement and frequent handling of poultry in LBMs create conducive conditions for virus spread. Environmental samples, including water, surfaces, and air, also tested positive for AIV, indicating widespread contamination within LBMs. Improving biosecurity protocols and promoting hygiene practices among market workers and visitors are essential for reducing AIV transmission and minimizing public health risks.

Our study provides crucial insights into the prevalence and diversity of AIV subtypes in Egyptian LBMs, emphasizing their potential risks. The higher prevalence of AIV in poultry intended for consumption compared to wild birds underscores a greater public health concern. The presence of highly pathogenic strains like H5N1 and H5N8 among poultry highlights the risk of severe illness and mortality, both in birds and possibly in humans. Similarly, the circulation of low pathogenic H9N2 in both poultry and wild birds raises concerns due to its potential for zoonotic transmission and its role in endemicity in poultry populations. Furthermore, the detection of diverse AIV subtypes in environmental samples indicates environmental contamination, posing additional challenges for disease control.

Migratory birds introduce new AIV strains into LBMs, especially during peak migration periods. While not directly assessed, the presence of wild birds in LBMs suggests their role in AIV introduction. LBMs facilitate AIV transmission due to diverse bird trading. Increased trading during festive seasons or high-demand periods contributes to higher AIV prevalence. Market activities like auctions and restocking also affect transmission dynamics. Poultry farming practices, market hygiene, and public health interventions impact AIV transmission. Changes in human behavior, like holiday market visits, influence AIV spread. Interventions, such as vaccination and biosecurity measures during specific seasons also affect AIV prevalence. Understanding human interventions is vital for interpreting seasonal trends in AIV transmission.

Our findings highlight the urgent need for implementing and enforcing robust biosecurity measures in LBMs to reduce the risk of AIV transmission. This includes measures such as restricting bird movement, proper waste management, disinfection protocols, and promoting hygiene practices among market workers and visitors. Future research should focus on assessing the effectiveness of specific biosecurity interventions in mitigating AIV transmission within LBMs. Comparative studies evaluating the impact of improved biosecurity measures on AIV prevalence and transmission dynamics would provide valuable insights for policy development and implementation. Additionally, collaborative efforts involving government agencies, veterinary services, public health authorities, and market stakeholders are essential for developing comprehensive strategies to control AIVs and prevent potential zoonotic transmission events.

## Conclusion

Our study identified diverse AIV subtypes, including highly pathogenic strains like H5N1 and H5N8, circulating among poultry, wild birds, and environmental samples. Temporal analysis revealed fluctuating positivity rates, indicating ongoing transmission dynamics. These findings underscore the importance of continuous surveillance, resource allocation, and collaborative efforts to prevent potential outbreaks and protect public health. Recommendations include implementing stringent biosecurity measures, enhancing environmental hygiene, and promoting One Health approaches to mitigate zoonotic transmission risks by applying One Health measures such as surveillance, outbreak investigation, multisectoral working groups, designing and implementing policies and strategies and fill critical gaps in scientific knowledge.

While our study provides valuable insights into the diversity of AIV subtypes within Egyptian LBMs, it’s essential to recognize the contextual factors unique to these markets and the Mediterranean coast cities where our study was conducted. The dynamics of AIV transmission can be influenced by various factors, including geographic location, climate, market practices, bird species composition, biosecurity measures, and human behavior. Therefore, caution must be exercised when extrapolating our findings to other regions or contexts with different characteristics. The presence of diverse AIV subtypes detected in our study, including H5N1, H9N2, H5/H9 co-infection, and H5N8, highlights the complexity of AIV circulation within Egyptian LBMs. However, the prevalence and distribution of these subtypes may vary in other geographical locations due to differences in poultry management practices, bird species composition, and environmental conditions. Therefore, it’s crucial to conduct similar surveillance studies in different regions to understand the regional variations in AIV epidemiology and inform context-specific control measures.

Furthermore, the seasonal variability in AIV positivity rates observed in our study may not be directly applicable to other regions with different climatic conditions and bird migration patterns. Factors such as temperature, humidity, and bird migratory routes can influence the timing and intensity of AIV transmission cycles. Therefore, caution should be exercised when extrapolating our findings to regions with distinct seasonal patterns.

The external validity of our results also depends on the representativeness of the LBMs sampled in our study. While we conducted surveillance in four LBMs located in Mediterranean coast cities, the dynamics of AIV transmission may differ in LBMs located in other parts of Egypt or in different countries. Therefore, future studies should aim to sample a more extensive range of LBMs across diverse geographic regions to enhance the generalizability of the findings. Future research efforts should focus on conducting surveillance studies in different geographic locations, considering local contextual factors, to improve the external validity and generalizability of the results. This approach will facilitate a better understanding of AIV epidemiology on a global scale and inform evidence-based control strategies tailored to specific regional contexts.

In conclusion, our study underscores the importance of surveillance, biosecurity, and collaborative efforts among stakeholders under One Health approach to prevent potentially dangerous new influenza strains and protect poultry, human, and environmental health.

## Data Availability

All data generated or analyzed during this study are provided within the manuscript.
