# Author Correction: Transmission dynamics of avian influenza viruses in Egyptian poultry markets

**DOI:** 10.1038/s44298-025-00130-z

**Published:** 2025-05-22

**Authors:** Sara H. Mahmoud, Mokhtar Gomaa, Ahmed El Taweel, Yassmin Moatasim, Mina Nabil Kamel, Mohamed El Sayes, Noura M. Abo Shama, Rebecca Badra, Mona Mahmoud, Pamela P. McKenzie, Richard J. Webby, Ahmed Kandeil, Mohamed Ahmed Ali, Rabeh El-Shesheny, Ghazi Kayali

**Affiliations:** 1 Center of Scientific Excellence for Influenza Viruses, National Research Centre, Giza, 12622 Egypt; 2 Human Link, Dubai, United Arab Emirates; 3 Department of Infectious Diseases, St. Jude Children’s Research Hospital, Memphis, TN 38105 USA; 4 Present Address: Texas Biomedical Research Institute, San Antonio, TX 78227 USA

Correction to: *npj Viruses* 10.1038/s44298-024-00035-3, published online 2 July 2024

“In the Acknowledgments section of the article, the correct funding contract number is HHSN75N93021C00016. This original article has been corrected”.

